# The Role and Limitations of Artificial Intelligence in Combating Infectious Disease Outbreaks

**DOI:** 10.7759/cureus.77070

**Published:** 2025-01-07

**Authors:** Hiba H Ali, Haya M Ali, Hera M Ali, Mohamad A Ali, Ahmed F Zaky, Anisa A Touk, Abdulkarim H Darwiche, Abdollfatah A Touk

**Affiliations:** 1 College of Medicine, Batterjee Medical College, Jeddah, SAU; 2 College of Medicine, University of Science and Technology, Irbid, JOR

**Keywords:** artificial intelligence, deep learning, infectious diseases, machine learning, neural networks, outbreak, pandemic, reinforcement learning

## Abstract

Artificial intelligence (AI) has emerged as a transformative tool in the management of pandemics, significantly enhancing disease prediction, diagnostics, drug discovery, and vaccine development. This manuscript explores AI's multifaceted applications during infectious disease outbreaks, from predictive modeling and outbreak forecasting to the acceleration of vaccine development and antimicrobial resistance detection. AI-driven technologies, including deep learning and reinforcement learning, have shown remarkable effectiveness in improving diagnostic accuracy, streamlining drug discovery processes, and providing real-time decision-making support for healthcare providers. However, despite its substantial contributions, the deployment of AI in pandemic management faces key limitations, including concerns about data privacy, model transparency, and the need for constant updates to adapt to emerging pathogens. The integration of AI with human expertise is essential to optimize global health outcomes and address these challenges. This review highlights both the potential and the obstacles to fully leveraging AI in pandemic response, proposing pathways for overcoming current limitations and maximizing AI’s impact on future outbreaks.

## Introduction and background

A pandemic is characterized as an infectious disease that spreads rapidly across vast geographical areas with very high transmission rates. Artificial intelligence (AI) has revolutionized the management of infectious diseases by enhancing various aspects such as disease prediction, diagnosis, drug and vaccine discovery, and treatment. A major advancement in AI is deep learning (DL), an advanced form of machine learning. DL utilizes complex neural networks to analyze extensive datasets, recognize patterns, and make predictions. Building on the capabilities of deep learning, another sophisticated technique is reinforcement learning (RL). RL mimics human decision-making by leveraging continuously updated data to provide tailored recommendations. RL adapts to individual patient situations and makes dynamic, real-time decisions [1-3].

This manuscript explores the applications and limitations of AI in the context of pandemics and infectious diseases, focusing on its role throughout all stages of an outbreak. AI has been instrumental in predicting, diagnosing, and treating infectious diseases, as well as aiding in vaccine and drug development. By integrating large datasets and continuously evolving algorithms, AI technologies can process vast amounts of information, offering unparalleled accuracy and efficiency. Despite these advancements, the full potential of AI in medical practice is contingent on addressing the limitations and challenges that currently exist, including issues surrounding data privacy, model transparency, and evolving pathogen strains.

## Review

Methodology

This systematic review aimed to investigate the role and limitations of artificial intelligence in pandemic management and infectious disease control. A structured literature search was conducted across multiple academic databases, including PubMed and Google Scholar, to identify relevant studies. Keywords such as "artificial intelligence," "machine learning," "pandemic management," and "infectious disease" were employed to ensure comprehensive coverage of the topic.

The search was restricted to English-language articles published from 2020 onward to focus on recent and relevant advancements. Studies were included if they specifically examined the application of AI in areas such as outbreak prediction, diagnostics, drug discovery, or vaccine development during pandemics. Articles that did not address AI’s role in pandemic management or infectious disease contexts were excluded.

Data extraction involved systematically collating information from the included studies, which was then synthesized to assess the impact of AI interventions. The findings provide insights into the effectiveness and challenges of AI in addressing pandemic-related issues.

Results

Role of AI Before the Pandemics

AI has demonstrated significant potential in outbreak forecasting and monitoring. By creating predictive models, AI supports efforts in contact tracing and surveillance. It integrates and analyzes diverse data sources, including social media activity, mobile phone data, flight records, public transportation data, individual mobility patterns, electronic health records, and genomic data. Through this analysis, AI effectively identifies clusters, high-risk areas, and outbreak hotspots [2].

Environmental changes, such as those driven by climate change - including rising sea levels and extreme weather events - have both direct and indirect impacts on community health. These shifts influence migration patterns, which, in turn, affect the spread and prevalence of infectious diseases, particularly vector-borne diseases [3]. AI’s ability to recognize and analyze ecosystem and environmental changes facilitates the prediction and management of such diseases [4].

Moreover, AI excels in processing large volumes of data with high accuracy. For instance, neural networks have been utilized to analyze electronic health records and genomic data, identifying individuals at increased risk for influenza by evaluating vital signs such as respiration rate, heart rate, and temperature [5]. Companies like BlueDot have successfully applied AI to predict the spread of diseases, including H1N1 in 2009, Ebola in 2014, and the Zika virus in 2019 [6,7].

The synergy between human expertise and AI-driven technologies is essential for preventing or mitigating the impacts of outbreaks and pandemics. By leveraging AI’s capabilities in data analysis and prediction, global health systems can enhance their preparedness and response to infectious disease threats.

Role of AI During the Pandemics

Increasing diagnostic accuracy: Diagnostic accuracy has significantly improved with the use of machine learning techniques. These tools help quickly, easily, and accurately identify patients and classify them accordingly. Convolutional neural networks (CNNs) have been successfully used to analyze medical images and identify certain diseases [8]. The Residual Network 50 (ResNet50) model, a type of CNN, has been used to analyze 1,752 COVID-19 chest X-ray (CXR) images, achieving an accuracy of 91% [9,10].

Support vector machines (SVM) are classifiers primarily utilized in tuberculosis cohorts, achieving 100% specificity, 100% sensitivity, zero root mean squared error (RMSE), 100% accuracy, and an area under the curve of one [11].

Drug discovery: The traditional process of drug discovery is complex and typically takes several years. However, involving AI in this process helps accelerate it, especially during pandemics [12]. AI has been applied at various stages of the drug discovery process. A critical step is target identification, with data sources such as gene expression profiles and protein-protein interaction networks used to compile a list of potential targets. This stage begins with modeling protein sequences using Modeller and AlphaFold 2. The protein sequence then undergoes homology modeling through ProQ and SolveX to validate its 3D structure. The next stage is pocket exploration, where the binding site is identified, including visible orthostatic pockets, allosteric pockets, and cryptic pockets.

In the hit identification stage, structure-based virtual screening (SBVS) and ligand-based virtual screening (LBVS) are used to identify potential compounds. The final stage involves toxicity prediction, where machine learning techniques assess absorption, distribution, metabolism, excretion, and toxicity properties of the drug [13]. A study presented a dataset called CoronaDB-AI, which can be used to train models in the future to discover a treatment for COVID-19 [14].

Detection of antimicrobial resistance: AI plays a crucial role in microbiology laboratories. During infectious diseases and outbreaks, it is crucial to identify antibiotic susceptibility in order to apply additional precautions or interventions to control the disease or prevent its spread. AI improves the accuracy and efficiency of identifying pathogens. For instance, machine learning-based matrix-assisted laser desorption ionization-time of flight mass spectrometry (MALDI-TOF MS) techniques have expedited the detection of methicillin-resistant Staphylococcus aureus (MRSA) and carbapenem-resistant Klebsiella pneumonia (CRKP), achieving an accuracy of 100% [15,16]. AFB+Neon Metafer is a module used to identify acid-fast bacilli on smear-negative slides, demonstrating significant accuracy and speed [16]. AI also enhances antimicrobial susceptibility testing. The SlipChip microfluidic device extracts bacteria directly from positive cultures and allows the parallel inoculation of bacteria, facilitating multiple antimicrobial susceptibility tests (AST) simultaneously [16].

Role of AI in Pandemic Recovery

Vaccine development: Vaccine discovery is crucial for both the prevention and recovery from infectious diseases and pandemics. Applying AI successfully accelerates the vaccine development process. AI has been instrumental in all stages of vaccine development. It can predict protein structures, such as antigens, which is the first step in the process. The second stage involves identifying or selecting adjuvants, which are essential for the vaccine’s effectiveness. Additionally, AI forecasts immune responses based on protein sequences. It can also analyze preclinical and clinical data to determine optimal booster doses and administration routes, enhancing the efficacy and safety of vaccines. Companies like Pfizer, AstraZeneca, and Moderna utilized AI at various stages of COVID-19 vaccine development [17,18].

## Conclusions

In conclusion, AI plays a pivotal role in pandemic management by enabling outbreak prediction, improving diagnostic precision, accelerating drug discovery, and advancing vaccine development. Additionally, it enhances clinical decision-making and supports the detection of antimicrobial resistance, contributing significantly to pandemic recovery efforts. However, despite these transformative benefits, the application of AI in pandemics faces notable challenges, including concerns about data privacy, lack of model transparency, and the need for regular updates to maintain relevance and accuracy. To maximize the potential of AI, its seamless integration with human expertise will be critical in optimizing pandemic response strategies and addressing these limitations effectively.
